# The dominance of leisure-time physical activity research: implications for health inequalities

**DOI:** 10.3389/ijph.2026.1609967

**Published:** 2026-08-28

**Authors:** Ragna Stalsberg

**Affiliations:** Department of Circulation and Medical Imaging, Faculty of Medicine and Health Sciences, Norwegian University of Science and Technology, Trondheim, Norway

**Keywords:** complex systems, distributive justice, equity, exercise, knowledge production, physical activity research

## Abstract

Physical activity research has substantially advanced our understanding of health benefits, yet it remains dominated by a narrow focus on leisure-time physical activity. This focus overlooks diverse physical activity practices in other domains and systematically misrepresents the experiences of low socioeconomic status groups. Methodological biases, including participant selection and physical activity assessment tools, further limit representativeness and weaken external validity, which contribute to epistemic injustice and persistent social health inequalities. This paper critically examines the conceptual, methodological, and ethical challenges in physical activity research that sustain inequities and argues for more inclusive, context-sensitive approaches. Adopting such approaches is essential for equitable health outcomes and to avoid reinforcing existing disparities through physical activity science.

## Background

Research on physical activity (PA) has received substantial funding due to its established benefits for health [1, 2] and longevity [3]. While researchers continue to advocate for investment in PA science to further understand its mechanisms and social profitability, it is essential to acknowledge some of its unintended social consequences, particularly those arising from how PA is conceptualized and studied.

Despite the well-documented health benefits of PA, and the many interventions that have followed, social health inequalities persist [4, 5]. Disparities between socioeconomic groups have often prompted the assumption that individuals with low socioeconomic status (SES) have poorer health because they are less physically active [6–8]. However, contemporary research indicates that the link between SES and PA is primarily within the domain of leisure-time PA and not in total PA as previously assumed [9–11]. This insight challenges our understanding of the PA–SES relationship and how PA should be conceptualized and researched. For example, public leisure-time PA interventions have a limited impact on overall social health inequalities, as they mostly benefit high-SES groups [11–13]. This dynamic is captured in the Inverse Care Law [14] and its subsequent adaptations [15]: those most in need derive the least benefit from such measures [16, 17].

Similar concerns appear in low-to middle-income countries, where researchers often conclude with a demand for leisure-time PA measures to address declines in PA and poor health [18, 19]. However, such recommendations account for neither the variety of PA domains, the distinction between necessity-based and choice-based PA [20], nor the structural complexity that affects SES inequalities [21]. In low-SES populations in low-to-middle-income countries, necessity-based occupational PA means that overall PA is often high [18, 22]. Accordingly, physical inactivity is unlikely to be a main cause of poor health, even in the context of increased levels of non-communicable diseases [23]. Such incongruities raise important concerns about PA research practices, particularly their role in perpetuating health inequalities due to embedded social biases [10, 23–25].

According to the principles of ethical practice in health research [26], researchers have a responsibility to uphold distributive justice through fair participant selection and equitable distribution of the burdens and benefits of research outcomes. One of the main principles is equity—that those with greater needs should receive more attention. PA research must, therefore, take the implications of the Inverse Care Law seriously by embedding distributive justice in research practice. Moreover, trustworthy research depends on reliable and valid propositions, inferences, and results. When what is considered valid knowledge is based on social bias—through conceptual framing, study design, and data collection—epistemic injustice emerges through systematic exclusion of the perspectives of certain social groups [27, 28].

Despite, the important contributions of PA research to understanding health and wellbeing, some approaches to PA research fall short in meeting these principles. To address the concerns outlined above, this paper offers an integrative analysis of conceptual, methodological, and assessment-related challenges that may (inadvertently) contribute to the reinforcement of social health disparities. The analysis adopts a methodological perspective and examines how research practices shape knowledge about PA across social groups. The central claim is that persistent social inequalities in PA are outcomes of unequal social conditions in addition to results of how PA research conceptualizes, measures, and represents PA across social groups.

## The PA concept: who defines what counts?

When high-SES researchers hold the authority to define what counts as PA, they favour leisure-time PA and marginalise necessity-based and utilitarian activities. This power imbalance distorts the evidence base, misrepresents low-SES practices, and undermines equity in knowledge production.

As a scientific concept, PA is defined by researchers working in academic environments influenced by high SES norms and values. Their own lifestyles often reflect these norms, which affect how they operationalise PA and potentially limit the representation of all SES groups [29].

PA is broadly defined as ‘*any bodily movement produced by skeletal muscles that results in [requires] energy expenditure* [30, 31]. The concept refers to all movements, including active transport, leisure-time activity, occupational activity [32], and domestic work. However, researchers face complexity in studying PA and must account for dimensions such as type, intensity, duration, frequency, and context while also balancing assessment feasibility and costs. In practice, these constraints often appear to result in a narrowing of the operational definition. Although most researchers acknowledge that PA includes more than physical exercise and leisure-time PA, and important research has examined occupational PA, domestic activities, avtive transport and sedentary behaviour [32, 33], several studies and guidelines continue to base their conclusions on leisure-time PA, often without explicitly stating this limitation [34, 35]. Hence, research framed as addressing PA in general draws conclusions about total PA, even though occupational PA, active transport, and household PA have been omitted from the assessments. This restricted understanding of PA also has an effect on public discourse, reinforcing a focus on leisure-time PA and overlooking SES-related differences in activity patterns. Empirical studies have shown SES-related differences in PA domains, with high-SES groups favouring leisure-time PA and low-SES groups engaging more in occupational and utilitarian forms of PA [9, 36, 37].

When health research and public policy focus primarily on leisure-time PA, low-SES groups are misrepresented. Their engagement in occupational or everyday PA is undercounted or ignored, creating the appearance of a PA deficit that may not exist. This misinterpretation helps explain why PA interventions targeting low-SES groups have limited success and why public health initiatives are generally less accepted in these populations [11, 13, 38, 39]. Designing interventions around high SES preferences may overlook low SES realities.

Common PA guidelines reinforce the same problem. By prescribing quantified targets—such as 150 min of moderate activity per week—such guidelines implicitly frame PA as structured and measurable behaviour. For many individuals, particularly those in lower SES groups, PA is embedded in necessary daily routines through work, caregiving, walking, or manual household tasks. These forms of activity are not as optional as leisure-time PA and labelling them as inadequate compared to leisure-time PA risks devaluing the physical exertion that low-SES groups already experience. Silva *et al.*, therefore, argue for a wider understanding of PA that includes utilitarian, necessity-based, and spontaneous forms of activity [40]. Recognising these forms is necessary for developing policies that reflect the diverse lived experiences of activity across SES groups. Although occupational and other necessity-based forms of PA may differ from LTPA in their health consequences [41, 42], and therefore warrant separate investigation, they remain important forms of PA and should therefore be adequately represented in PA research.

## Study designs: whose data matter?

When PA trials rely on structured exercise protocols and strict eligibility criteria, they embed social bias that excludes low-SES experiences. These patterns compromise external validity and perpetuate inequities in evidence-based practice.

Substantial evidence supports the health benefits of PA and leads to increased clinical trials investigating the effects of specific PA programs on health outcomes. However, strict inclusion and exclusion criteria, along with attrition bias [43, 44], limit the real-world relevance and generalisability of these studies [45]. Inclusion biases occur when participants are disproportionately active at baseline or predominantly recruited from higher SES groups. Moreover, social attrition bias follows when participants’ adherence to prescribed PA regimens is influenced by SES, further undermining representativeness and skewing conclusions due to a lack of representative data for some SES groups.

PA interventions in clinical trials are commonly designed with detailed exercise protocols. However, given that high-SES groups engage primarily in leisure-time PA, while low-SES groups are more active through occupational forms of PA [9, 36], these programs appear unattractive, impractical, or irrelevant for low-SES populations. This mismatch reduces recruitment willingness [46, 47], and adherence among low-SES participants. In addition to these challenges, many trials fail to report participants’ SES and thereby limiting the assessment of sample representativeness [48, 49]. Systematic reviews confirm these trends, for example, in breast cancer PA trials [50], and similar patterns emerge in other clinical contexts. Although data on presurgical exercise adherence among older adults is limited [51], studies on cardiac rehabilitation indicate poorer adherence among low-SES patients compared to their high-SES counterparts [52]. Reports of no SES differences in adherence in some trials may therefore reflect misrepresentation rather than true balance [53, 54].

Reporting practices can further distort representativeness. Many randomised clinical trials report adherence only among participants who complete the intervention, while overlooking those who withdraw, and significantly skewing the results [55, 56]. To report only on completers distorts the actual representation of the target patient group and withholds valuable information and potentially conceals limitations in the intervention’s feasibility and obscures social patterns among dropouts and low-adherence participants.

The above points reflect a stepwise and cumulative social exclusion process in PA trials. Initial refusal to participate, criteria-based pre-trial exclusion, midtrial dropouts for various reasons, including regret over participation, and poor adherence, eliminate low-SES experience from the evidence base. Importantly, these patterns are likely influenced by broader social circumstances that shape opportunities and willingness to participate in research, but may also be reinforced by recruitment practices, study designs, and intervention requirements. If such exclusion is socially patterned throughout the research process, the external validity progressively diminishes.

These cumulative exclusions do more than weaken external validity—they shape the evidence base that informs policy and practice. When interventions and services are built on research that disproportionately reflects high SES preferences, they risk reinforcing health disparities rather than reduce them.

## PA assessments: which data matter?

Assessment tools that prioritise quantification of leisure-time PA obscure the complexity of activity patterns across SES groups. This omission distorts scientific understanding and limits the development of equitable interventions.

A variety of tools exist for measuring PA, determined by the objectives and epistemological approach of the researcher, yet none is considered a gold standard. Because the health effects of PA are linked to its quantified volume [57], most assessments necessarily rely on quantification. Self-report surveys remain the most common method for estimating PA levels [9, 58–60], likely due to their low cost and convenience. However, these instruments suffer from well-documented limitations [59–62], and are rarely developed or validated for individuals with chronic conditions or disabilities that limit regular PA participation [63].

Repeated use of the same instruments produces uniform, quantified results, reinforcing assumptions about PA rather than revealing its complexity. When questionnaires frame activity consistently in terms of leisure-time PA, they fail to capture the qualitative differences in experiences, preferences, and constraints across SES groups. These omissions risk misrepresenting the activity patterns of lower SES populations, particularly since health conditions and sociodemographic factors often lead to irregular PA in these groups [62]. Such shortcomings can result in serious distortions of quantified PA levels and interventions that overlook actual practices. Evidence from mixed-methods research points to the need for diverse methodologies to capture these variations and design more equitable interventions [64].

## Epistemic injustice: when social bias influences knowledge production

When social bias affects what counts as valid knowledge, PA research risks committing epistemic injustice—systematically excluding certain perspectives and misrepresenting lived realities.

The methodological shortcomings outlined in the previous sections do not merely affect validity; they determine what counts as knowledge. Epistemic injustice occurs when certain social groups are systematically disadvantaged in their ability to contribute to or access knowledge [27]. In PA research, this injustice appears through socially patterned biases in conceptual framing, study design, and data collection. The narrow operationalization of PA, combined with methodological practices that favour leisure-time PA, creates a knowledge base that disproportionately reflects the experiences and activity patterns of higher SES groups while giving less visibility to PA practices that are more common among low-SES populations. This constitutes a form of hermeneutical injustice in which dominant conceptual frameworks obscure or devalue alternative experiences and practices [28].

Social bias in research practices can also lead to testimonial injustice, where the credibility of certain groups is systematically underestimated. For example, when assessment tools and study design primarily focus on leisure-time PA, the experiences of individuals whose activity occurs mainly through work, caregiving, or other daily obligations become less visible in the evidence base. Consequently, knowledge about PA is more strongly formed by some experiences han others. These patterns are reinforced by data collection tools and evidence hierarchies that favour standardised, quantifiable measures over context-sensitive approaches [65]. Such practices both distort scientific understanding and perpetuate inequities in health interventions, as policies informed by biased evidence fail to meet the needs of those most affected by health disparities.

## Persistent inequalities as emerging effects: sustainable research practices?

If PA research perpetuates epistemic injustice, its effects accumulate into persistent social health inequalities—raising questions about the sustainability and ethics of current research practices.

The epistemic injustices described above do not operate in isolation; they interact with policy decisions and social structures, creating reinforcing cycles of exclusion. Understanding these patterns as emergent effects of complex systems is essential for developing sustainable research practices.

Jones et al. [66] argue that inequalities in PA should be understood as dynamic features of complex social systems arising from nonlinear interactions among research practices, policy decisions, and social structures over time. These interactions aggregate into persistent patterns of exclusion and inequity that cannot be explained by isolated variables alone.

Importantly, these reinforcing cycles are also epistemically consequential. When biased research practices define what counts as valid evidence, they perpetuate the hermeneutical and testimonial injustices discussed earlier—further marginalizing low-SES perspectives. This epistemic distortion feeds directly into policy and intervention design, creating a feedback loop that privileges high-SES norms while neglecting other forms of PA.

The dominant focus on leisure-time PA in PA research produces a reinforcing feedback loop: Studies emphasise leisure-time PA or exercise, which in turn guide policies, interventions, and infrastructure investments that serve high-SES groups. These groups benefit from such initiatives, which, in turn, appear to validate the continued emphasis on leisure-time PA and justify further investment. Meanwhile, the PA practices of low-SES populations remain underrepresented, undervalued, and underserved [20, 40].

This research-policy cycle is both limiting in terms of knowledge production and raises important ethical concerns. The misrepresentation of low-SES groups risks reinforcing stigma when findings based primarily on leisure-time PA are interpreted as reflecting PA more broadly. In such cases, individuals may appear insufficiently active despite engaging in substantial amounts of occupational, domestic, or other necessity-based forms of activity. This may direct attention towards individual behaviour rather than the social and structural conditions that shape activity patterns. From a sustainability perspective, these patterns carry multiple consequences. Economically, reiterative research that fails to address the needs of low-SES populations may result in inefficient use of public resources [17]. Socially, privileging high-SES-targeted interventions—either in research or policy—exacerbates health disparities and undermines the principles of equity [16]. Environmentally, the promotion of leisure-time PA or exercise—often through car-dependent recreational infrastructure and costly equipment—may conflict with wider sustainability goals. In contrast, utilitarian forms of PA may not only be more relevant to low-SES groups but also contribute to climate-friendly urban development.

### Conclusion

This conceptual analysis demonstrates how prevailing practices in PA research contribute to persistent social health inequalities. The dominance of high-SES perspectives in defining and measuring PA, the narrow emphasis on leisure-time PA, and recurring methodological exclusions undermine both the relevance and fairness of the evidence base. These shortcomings not only compromise the accuracy of scientific knowledge. They also stimulate to interventions that fail to reach those most affected by health disparities. In addition to methodological validity and social justice, these challenges are fundamentally epistemic: When social bias guides conceptualization, study design, and data collection, epistemic injustice emerges—systematically excluding perspectives and misrepresenting lived realities. Addressing these concerns requires deliberate efforts to revise the conceptual framework, diversify methodologies, and explicitly confront epistemic injustice. Examples include the use of assessment tools that capture multiple PA domains, greater use of mixed-methods approaches, more systematic reporting of socioeconomic characteristics, and study designs that better accommodate the realities and constraints faced by low-SES populations. Such efforts are essential to ensure that knowledge production is sustainable, equitable, and responsive to all socioeconomic groups.
